# Supplementary material for: Netcombin: An algorithm for constructing optimal phylogenetic network from rooted triplets
Source: PLoS One. 2020 Sep 18;15(9):e0227842. doi: 10.1371/journal.pone.0227842 (PMC7500971; doi:10.1371/journal.pone.0227842)
Supplement: S4 File — (ZIP) [file pone.0227842.s004.zip › SequenceData-Triplet/sequenceData-10-20-30-0-Triplet/length 200, numseq 40, rate 10^-7/6 seq.pdf]

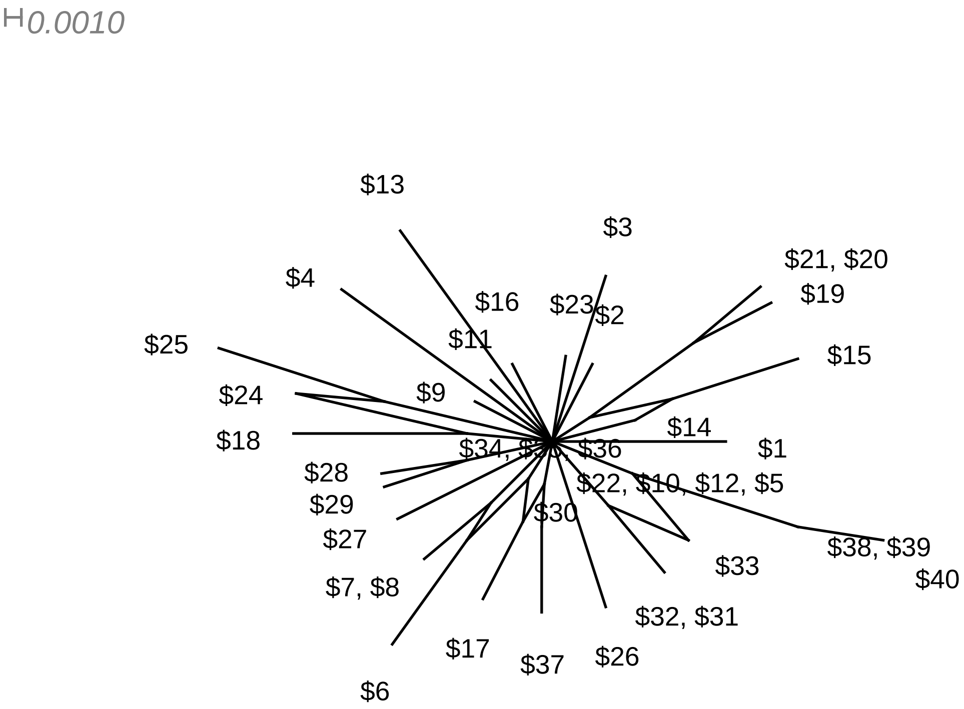
0.0010
$13
$3
$21, $20
$4
$16
$23
$2
$19
$25
$11
$15
$24
$9
$14
$18
$34,
$36
$1
$28
$22, $10, $12, $5
$29
$30
$27
$38, $39
$33
$40
$7, $8
$32, $31
$17
$37
$26
$6
